# Phenolic Fractions from Dandelion Leaves and Petals as Modulators of the Antioxidant Status and Lipid Profile in an In Vivo Study

**DOI:** 10.3390/antiox9020131

**Published:** 2020-02-03

**Authors:** Michał Majewski, Bernadetta Lis, Jerzy Juśkiewicz, Katarzyna Ognik, Małgorzata Borkowska-Sztachańska, Dariusz Jedrejek, Anna Stochmal, Beata Olas

**Affiliations:** 1 Department of Pharmacology and Toxicology, Faculty of Medicine, University of Warmia and Mazury in Olsztyn, 10-082 Olsztyn, Poland; 2 Department of General Biochemistry, Faculty of Biology and Environmental Protection, University of Łódź, 90-236 Łódź, Poland; bernadetta.lis@biol.uni.lodz.pl (B.L.); beata.olas@biol.uni.lodz.pl (B.O.); 3 Division of Food Science, Institute of Animal Reproduction and Food Research of the Polish Academy of Sciences, 10-748 Olsztyn, Poland; j.juskiewicz@pan.olsztyn.pl; 4 Department of Biochemistry and Toxicology, Faculty of Biology, Animal Sciences and Bioeconomy, University of Life Sciences, 20-950 Lublin, Poland; kasiaognik@poczta.fm; 5 Department of Psychiatry, Faculty of Medicine, University of Warmia and Mazury in Olsztyn, 10-228 Olsztyn, Poland; malgorzata.borkowska@uwm.edu.pl; 6 Department of Biochemistry and Crop Quality, Institute of Soil Science and Plant Cultivation, State Research Institute, 24-100 Puławy, Poland; djedrejek@iung.pulawy.pl (D.J.); asf@iung.pulawy.pl (A.S.)

**Keywords:** chicoric acid, dandelion, hydroxycinnamic acids, oxidative stress, *Taraxacum officinale*

## Abstract

Alcoholic leaf and petal fractions of *Taraxacum officinale* (dandelion) were previously demonstrated to exert in vitro antioxidant and antithrombotic activities in blood plasma and platelets. Eight-week-old male Wistar rats (*n* = 6) were supplemented for four weeks with dandelion fractions (694 mg/kg of diet = 11.9 ± 0.6 mg daily). Dandelion leaf and petal fractions, which delivered daily 4.10 ± 0.05 and 1.41 ± 0.07 mg l-chicoric acid, respectively, were shown to exert antioxidative actions, measured as decreased levels of thiobarbituric acid-reactive substances (TBARS) in the spleen (≈0.8-fold, leaves and petals), brain (0.53-fold, leaves) and thoracic arteries (0.59-fold, petals). Moreover, petal fraction increased thiols in the blood plasma (1.58-fold), while leaf fraction decreased protein carbonylation levels (0.59-fold). Additionally, dandelion leaf fractions modified the lipid profile: decreased triglyceride (0.44-fold), total cholesterol (0.73-fold), lipoprotein combine index (0.32-fold) and the atherogenic index of plasma (0.62-fold). Dandelion fractions showed a beneficial decrease effect in the participation of cyclooxygenase products in the noradrenaline-induced vascular contractions of thoracic arteries. Meanwhile, only the dandelion leaf fraction augmented acetylcholine-induced vasodilation and upregulated K_ATP_ channels. The heart rate and blood pressure were not modified. Dandelion leaf and petal phenolic fractions, enriched with l-chicoric acid, are promising plant materials that may exert in vivo beneficial antioxidant effects.

## 1. Introduction

Oxidative stress associated with the presence of an excess of pro-oxidants, including free radicals, can cause oxidative modifications of lipids and proteins in the components of the hemostatic system (blood plasma and platelets) and can increase its reactivity (increased clotting). Such changes in vascular hemostasis contribute to various pathological conditions of the cardiovascular system, such as thrombosis and atherosclerosis. Thus, preparations exerting both antioxidant and anticoagulant activities based on safe natural substances are continuously sought after for their potentially wide range of health benefits in the prevention and treatment of cardiovascular disorders [1,2,3,4,5,6,7].

Nowadays, the dandelion (*Taraxacum officinale*) herb and root are used as a component of food products, dietary supplements and pharmacological preparations for various ailments (mainly liver, gallbladder and kidney disorders) [8,9]. This is due to a wide range of biological activities, including antihyperglycemic, antioxidant and anti-inflammatory actions demonstrated by dandelion fractions and their constituents, with no reported adverse effects, together with a well-understood chemical composition and a widespread occurrence of the plant throughout the world [10,11]. Aqueous-organic extracts from the dandelion herb were found to exert an anti-inflammatory action [12], mainly due to decreasing the expression of proinflammatory cytokines by inhibiting the nuclear factor (NF)-κB [13], tumor necrosis factor (TNF)-α and interleukin (IL)-1β [14], as well as reducing the expression and production of inducible nitric oxide synthase/nitric oxide (iNOS/NO) and cyclooxygenase (COX)-2/prostaglandin E2 [15].

Plant polyphenols have the ability to protect dietary lipids, proteins and vitamins from oxidation. Additionally, they may also provide health benefits associated with preventing damage to the building elements of the body (DNA, lipids and proteins) due to decreased biological degeneration [16,17,18]. A number of polyphenolic compounds, mainly phenolic acids (hydroxycinnamic acid (HCAs) derivatives) and flavonoids (apigenin and luteolin derivatives) have been identified in the leaves and flowers of dandelions, so far [19,20,21]. Natural bioactive substances with both antioxidative and hypo-cholesterolemic properties, an example of which is chicoric acid, have been found to be effective in preventing the formation and/or progression of atherosclerosis [18,22]. HCAs induce antiradical and protective actions against oxidation processes [3,21,23]; meanwhile, flavonoids inhibit the formation of reactive oxygen and/or nitrogen species by suppressing NO synthase and COX-2 protein expression [16,17,24]. 

In our previous in vitro studies, the standardized alcoholic fractions from petals and leaves of *T. officinale* (including HCAs and flavone fractions) were found to possess the ability to reduce oxidative stress (i.e., lipid peroxidation, protein carbonylation and protein thiol groups) in human plasma and blood platelets [3,21]. Additionally, dandelion fractions enriched with HCAs, derived from both leaves and petals, were shown to beneficially influence the coagulation activity of blood plasma [3].

With increasing evidence of the protective potential of dandelion fractions on the antioxidant status but with scarce data concerning its properties in the systemic vasculature, we aimed to examine whether dandelion fractions from leaves and petals influence, in the same way (i) the antioxidant profile (of internal organs, blood plasma and urine samples); (ii) modify the blood plasma lipids; (iii) glucose and (iv) the intestinal digesta. In addition, we analyzed (v) the reactivity of isolated rat thoracic arteries, and (vi) we measured the heart rate (HR) and blood pressure (BP) with the tail-cuff system in Wistar rats.

## 2. Materials and Methods

### 2.1. Plant Material

The leaves and flowers of *T. officinale* were harvested on a farm located in south-eastern Poland (Rzeszów, 50.114175° N, 21.911738° E) in 2015. Dandelion material was freeze-dried, powdered and used for extraction, as previously described [21].

#### Extraction and Preparation of Dandelion Phenolic Fractions

Briefly, the defatted dandelion leaves and petals were first extracted with alcohol (methanol 80%) under reflux. The subsequently obtained raw leaf and petal extracts were evaporated and loaded onto a short glass column filled with Cosmosil C18-PREP 140 (140 μm, 6 × 10 cm, Nacalai Tesque Inc., Kyoto, Japan). To remove carbohydrates, column was washed with water and then with methanol (50%) to elute phenolic compounds. Polyphenols present in both fractions (leaves and petals) were tentatively characterized and quantified using the LC-PDA-ESI-MS/MS technique [21].

### 2.2. Drugs and Chemicals

The drugs used were: acetylcholine (ACh) as chloride, indomethacin (Indo), N(ω)-nitro-l-arginine methyl ester (l-NAME) hydrochloride, noradrenaline (NA) as hydrochloride, sodium nitroprusside (SNP), tricarbonyldichlororuthenium (II) dimer (CORM-2) (Sigma-Aldrich, Schnelldorf, Germany) and pinacidil (Cayman Chemical, Hamburg, Germany). Indomethacin was dissolved in ethanol. NA was dissolved in a mixture of sodium chloride + ascorbic acid (0.9% and 0.01% *w*/*v*, respectively). Other drugs were prepared in distilled water. The stock solutions (10 mM) were maintained at −20 °C, and appropriate dilutions were made in Krebs-Henseleit solution (KHS; mM; NaCl 115, CaCl_2_ 2.5, KCl 4.6, KH_2_PO_4_ 1.2, MgSO_4_ 1.2, NaHCO_3_ 25 and glucose 11.1 at pH 7.4) on the day of the experiment.

Cytochrome c, dimethyl-sulfoxide (DMSO), formic acid, hydrogen peroxide and thiobarbituric acid of LC-MS grade were purchased from Sigma Aldrich (St. Louis, MO, USA). Methanol (isocratic grade) and acetonitrile (LC-MS grade) were acquired from Merck (Darmstadt, Germany). All other reagents were of analytical grade and were provided by commercial suppliers.

### 2.3. Ethics Statements

The study was approved by the Local Ethics Committee for Animal Experimentation in Olsztyn, Poland (Permission No. 67/2018) and was performed in accordance with the European Union Directive 2010/63/EU for animal experiments and conformed to the *Guide for the Care and Use of Laboratory Animals* (US National Institutes of Health Publications No. 86–26, revised 2014). All efforts were made to minimalize animal suffering. The replacement, reduction and refinement (3Rs) rule was respected in the study.

### 2.4. Animal Protocol and Dietary Treatment

Male albino Wistar rats [Han IGS rat (Crl:WI(Han))] at 8 weeks of age were randomly divided into three groups of 6 animals each. Group 1 (the control group) was not supplemented with dandelion. Group 2 and group 3 were fed with a diet supplemented with either dandelion leaf or petal fractions (694 mg/kg of diet = 11.9 ± 0.6 mg daily) for a period of 4 weeks.

Rats were kept individually in stainless steel cages under the following conditions: temperature of 21–22 °C, a relative humidity of 50 ± 10% and a ventilation rate of 20 air changes during one hour. The rats had free access to tap water and 20 g per day of experimental diets, which were prepared weekly and then stored at 4 °C in hermetic containers until the end of the experiment. The experimental diets were modifications of a casein diet recommended by the American Institute of Nutrition for laboratory rodents.

### 2.5. Experimental Procedures

Rats were anesthetized by intraperitoneal injection of ketamine (100 mg/kg BW) and xylazine (10 mg/kg BW) and killed by decapitation. Blood samples were kept in tubes containing heparin + EDTA as an anticoagulant and centrifuged at 3000× *g* for 10 min to separate the blood plasma, which was stored at −80 °C until further analysis. The organs such as brain, heart, liver, spleen, thoracic aorta and intestine (small intestine and cecum) were carefully isolated and weighed.

#### 2.5.1. Blood Plasma Glucose and Lipid Profile

The content of glucose, triglyceride (TG), total cholesterol (TC), high-density lipoprotein cholesterol (HDL-C) and low-density lipoprotein cholesterol (LDL-C) were measured using a biochemical auto-analyzer (Horiba, Kyoto, Japan). The results were expressed as mmol/L.

#### 2.5.2. ORAC, TBARS, Thiol and Carbonyl Groups

Blood plasma antioxidant capacity was evaluated by oxygen radical absorbance capacity (ORAC) fluorescence assay, as described earlier [25]. The results were expressed as mmol TE/L.

To measure thiobarbituric acid-reactive substances (TBARS), samples of blood plasma and organ homogenates were mixed with an equal volume of 15% (*w*/*v*) cold trichloroacetic acid in 0.25 M HCl and with an equal volume of 0.37% (*w*/*v*) thiobarbituric acid in 0.25 M HCl. Samples were then immersed in a boiling water bath for 10 min. After cooling and centrifugation, absorbance was measured at 535 nm, and results were expressed as nmoles of TBARS per mL of plasma or per mg of proteins [3,21].

The oxidation of amino acid residues in proteins was measured by determining the amounts of carbonyl and thiol groups. The detection of protein carbonyls involved derivatization of the carbonyl groups with 2,4-dinitrophenylhydrazine (DNPH), which lead to the formation of a stable 2,4-dinitrophenyl (DNP) hydrazone product, as described previously [26].

The total thiol group content was measured spectrophotometrically (the absorbance at 412 nm; the SPECTROstar Nano Microplate Reader-BMG LABTECH, Ortenberg, Germany) with Ellman’s reagent 5,5′-dithio-bis-(2-nitrobenzoic acid), as described previously [3,21]. The results were expressed as nmol/mg of tissue homogenate and nmol/mL/mg of protein.

The protein concentration was calculated by measuring the absorbance of tested samples at 595 nm, according to the procedure of Bradford [27].

#### 2.5.3. The Intestinal Digesta

The pH values of the small intestine, cecum and colon digesta were measured directly in the intestine segments with a pH meter (model 301, Hanna Instruments, Vila do Conde, Portugal).

#### 2.5.4. Urine F2α-Isoprostane

Urine samples were stored at −80 °C, and the concentration of 8-isoPGF2α was examined with an immunoassay kit (Cayman Chemical, Ann Arbor, MI, USA) in accordance with the manufacturer’s instructions.

#### 2.5.5. Blood Pressure and Heart Rate in Rats

On the day before blood collection, the rats underwent HR and BP monitoring. These were measured using the noninvasive blood pressure system (tail-cuff, LE5001, Panlab, Harvard Apparatus, Barcelona, Spain) in conscious rats. HR and BP were calculated as the average of eight consecutive readings.

#### 2.5.6. Vascular Reactivity Studies

The thoracic arteries were cleaned of adherent tissue, cut into 6–8 rings and suspended horizontally under a resting tension of 1 g in 5-mL tissue baths (Graz, Harvard Apparatus, Barcelona, Spain) containing KHS, aerated with a carbogen (95% oxygen and 5% carbon dioxide), maintained at 37 °C and at pH of 7.4 [7]. Each ring was connected with a transducer and amplifier (F-30, TAM-A Hugo-Sachs Elecktronik, March, Germany) to measure the isometric force.

Aortic rings were washed 3 times in KHS over 60 min and exposed to NA (0.1 µM) that induced approximately 1 g of contraction. Next, the cumulative concentrations of ACh (0.1 nM–10 µM); NO donor sodium nitroprusside (SNP, 0.1 nM–10 µM); carbon monoxide (CO) donor tricarbonyldichlororuthenium (II) dimer (CORM-2, 1–100 µM) and ATP-sensitive K^+^ channel (K_ATP_) opener pinacidil (10 nM–10 µM) were added to assess the endothelium-dependent vasodilation.

In another set of experiments, the NO synthase inhibitor l-NAME (100 μM) and the nonspecific COX inhibitor indomethacin (Indo, 10 μM) were added 30 min before the contraction with NA was performed.

### 2.6. Data Analysis and Statistics

A nontraditional lipid profile was calculated as the atherogenic index of plasma (AIP): log_10_(TG/HDL-C), lipoprotein combine index (LCI): TG*TC*LDL-C/HDL-C, atherogenic index (AI): non-HDL-C/HDL-C, LDL-C/HDL-C, TC/HDL-C and as non-HDL-C: TC minus HDL-C.

The calculations and graphs were done and analyzed in GraphPad Prism 8.3. The NA-induced contraction was expressed in mg of developed tension. Vascular relaxation was expressed as a percentage of the response to NA (0.1 µM). The individual CCRCs were analyzed by nonlinear regression model, which determined the maximal response (E_max_, %) and the potency (the negative logarithm of the concentration causing a half-maximum effect, pD_2_).

The Gaussian distribution of residuals and homoscedasticity of variance were tested for all data. The group comparison was performed by two-way ANOVA with Tukey’s post hoc test. The graphs present results as mean ± SD, whereas Tables S1–S3 express results as mean ± SEM and median (with the 25th and 75th percentiles) from *n* = 6 rats per each group. Due to limitations on sample volume collected from animal subjects and/or data outlier detection by the Grubbs’ test, *n* varies among bioassays. Differences were considered significant when *p* ≤ 0.05.

## 3. Results

### 3.1. The Phytochemical Characteristics of Dandelion Leaf and Petal Fractions

The chromatographic analyses of dandelion leaf and petal fractions revealed that the HCAs, especially esters of caffeic acid, were the dominant polyphenolic compounds present in the analyzed fractions. In the leaf fraction, the total amount of HCAs was equal to 420 mg/g dry weight (DW) (the main component was l-chicoric acid at about 350 mg/g DW ≈ 83%), whereas in the petal fraction, the total amount of HCAs was equal to 214 mg/g DW (the main component was l-chicoric acid at about 117 mg/g DW ≈ 55%). These fractions delivered daily 4.10 ± 0.05 mg and 1.41 ± 0.07 mg of l-chicoric acid per rat, respectively. A more detailed characterization of the above fractions has been previously described [21].

### 3.2. General Characterization of Rats

Leaf and petal fractions from *T. officinale* neither affected the animal weights (*p* ≥ 0.2516) nor the dietary intake (*p* ≥ 0.3722) during the four weeks of feeding.

The mass of internal organs-to-body weight ratio (expressed in %) and the intestinal samples did not differ between the analyzed groups (all values of *p* ≥ 0.1760). Data are summarized in Table S1.

### 3.3. The Intestinal Digesta

The mass of the cecum and the small intestine (*p* ≥ 0.1442), as well as the pH of the cecum content (*p* ≥ 0.2260), were not modified in a significant way; see Table S1.

### 3.4. Blood Pressure and Heart Rate in Rats

Dandelion leaf and petal fractions had no significant effect on BP (*p* ≥ 0.1634) and HR (*p* ≥ 0.9916) in Wistar rats; see Table S1.

### 3.5. Blood Plasma Glucose and Lipid Profile

**Figure 1:**
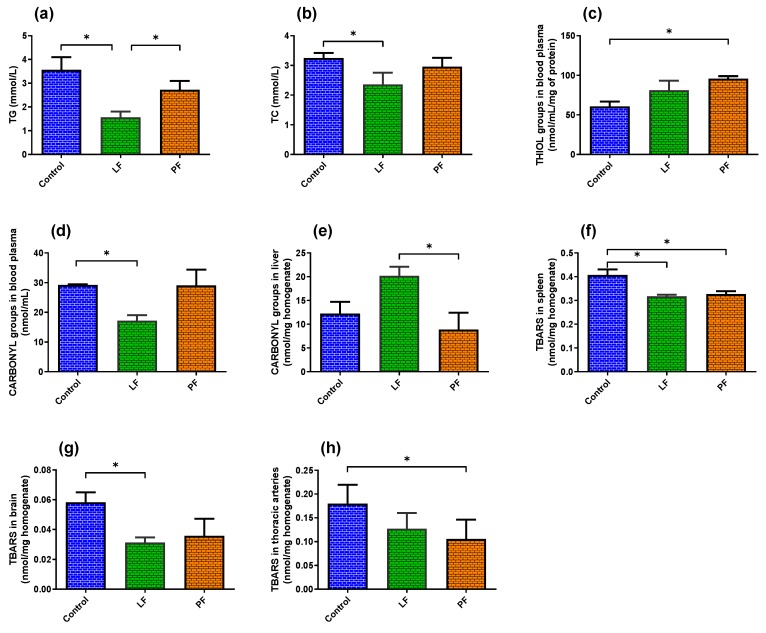
The significant impact of experimental diets on (**a**) triglycerides, (**b**) total cholesterol, (**c**) thiol groups, (**d**,**e**) carbonyl groups and (**f**–**h**) TBARS. From the 8^th^ week of age, rats were supplemented for 4 weeks with either dandelion leaf fraction (LF) or petal fraction (PF). The control group was not supplemented with dandelion. Results are expressed as mean ± SD, *n* = 6. * *p* ≤ 0.05 (two-way ANOVA/Tukey’s); see supplementary materials. Abbreviations: TBARS, thiobarbituric acid reactive substances; TG, triglycerides and TC, total cholesterol.

The tested fractions of *T. officinale* decreased the TG to 0.44-fold (*p* = 0.0262, leaf fraction) and 0.76-fold (*p* = 0.2634, petal fraction), compared to the control group. However, only in the leaf fraction was the effect statistically significant (Figure 1a). A significant 0.57-fold decrease of TG was also observed in the dandelion leaf fraction, compared to petal fraction (*p* = 0.05, Figure 1a). The leaf fraction caused a significant decrease in the level of blood plasma TC of a 0.73-fold decrease, compared to the control group (*p* = 0.05, Figure 1b). Additionally, the nontraditional lipid profile calculated as LCI (0.32-fold, *p* = 0.05) and AIP (0.62-fold, *p* = 0.0449) was decreased in tested leaf fractions in comparison to the control group (Table S2).

The blood plasma glucose, HDL-C, LDL-C, non-HDL-C, TC/HDL-C, LDL-C/HDL-C and AI did not change in a significant way (all values of *p* ≥ 0.1345). Data are summarized in Table S2.

### 3.6. The Antioxidant Status

Both dandelion leaf and petal fractions increased the number of thiol groups in blood plasma, compared to the control group (1.34- and 1.53-fold, respectively). Nevertheless, only in the petal fraction was the effect statistically significant (*p* = 0.0076 vs. *p* = 0.2033); see Figure 1c.

In contrast, only the leaf fraction decreased the plasma protein carbonylation level (0.59-fold decrease in comparison to the control, *p* = 0.0214); see Figure 1d. The same leaf fraction showed a rather pro-oxidative effect in rat organ homogenates: liver (*p* = 0.0646, Figure 1e); heart; spleen and brain (*p* ≥ 0.3636) trends without statistical significance (Table S3). Interestingly, a significant 0.57-fold decrease in carbonyl groups was observed in liver homogenates of rats supplemented with petal fractions, compared to rats supplemented with leaf fractions (*p* = 0.0486, Figure 1e).

Tested dandelion fractions had neither a strong influence on the plasma antioxidant capacity, measured as ORAC (*p* ≥ 0.091), nor on the markers of lipid peroxidation in blood plasma, heart and liver homogenates, measured by the level of TBARS (*p* ≥ 0.1706, Table S3). Meanwhile, analyzed fractions did exert a slight protective effect on lipids (decreased TBARS) in the spleen (leaf: 0.78-fold, *p* = 0.0208 and petal: 0.80-fold, *p* = 0.0379; Figure 1f); brain (leaf: 0.53-fold, *p* = 0.0225 and petal: 0.62-fold, *p* = 0.1651; Figure 1g) and thoracic arteries (leaf: 0.71-fold, *p* = 0.05 and petal: 0.59-fold, *p* = 0.05; Figure 1h). Data are summarized in Table S3.

### 3.7. Urine F2α-Isoprostane

No significant differences were observed in 8-isoPGF2α levels between dandelion fractions and the control group (*p* ≥ 0.5025, Table S3).

### 3.8. Vascular Reactivity Studies

**Figure 2:**
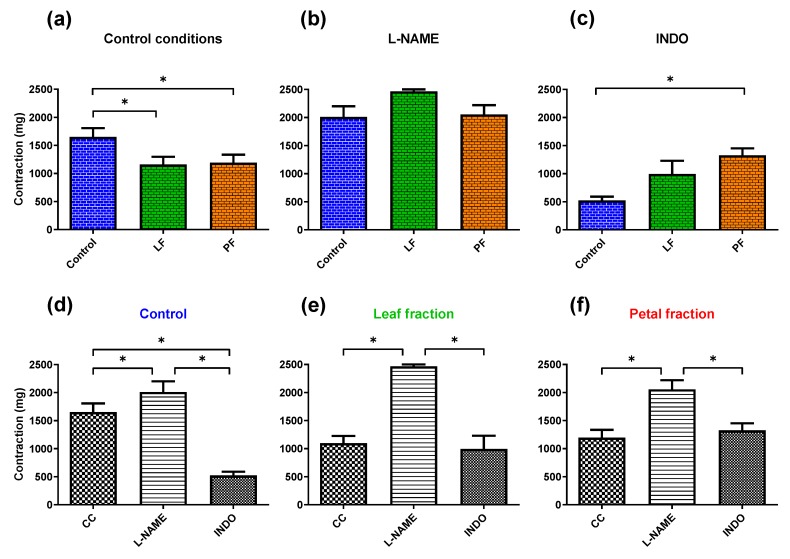
Contractile response of rat thoracic arteries to noradrenaline (NA, 0.1 μM) (**a**–**f**). The effect obtained under (**a**) control conditions; (**b**) with nitric oxide synthase inhibitor (l-NAME, 100 μM, 30 min) and (**c**) with the nonselective cyclooxygenase inhibitor (indomethacin, 10 μM, 30 min) on NA-induced contractions. From the 8^th^ week of age, rats were supplemented for 4 weeks with either dandelion leaf fraction (LF) or petal fraction (PF). The control group was not supplemented with dandelion. Results are expressed as mean ± SEM, *n* = 6. * *p* ≤ 0.05 (two-way ANOVA/Tukey’s). Abbreviations: CC, control conditions; Indo, Indomethacin and l-NAME, N(ω)-nitro-l-arginine methyl ester.

The contractile response generated by a single dose of NA (0.1 μM) was decreased in aortic rings from rats supplemented with dandelion petal and leaf fractions by 0.72- and 0.66-fold (*p* ≤ 0.036), respectively; see Figure 2a. Preincubation with NO synthase inhibitor l-NAME (100 μM, 30 min) did not modify the contractile response between the experimental groups (Figure 2b). Preincubation with the nonselective COX inhibitor indomethacin (10 μM, 30 min) did enhance the NA-mediated contraction in rats supplemented with both phenolic fractions; nevertheless, the effect was significant only in the case of petal fractions (2.52-fold increase compared to the control group, Figure 2c). In the control group, preincubation with indomethacin decreased to 0.32-fold the contraction, as compared with the control conditions (*p* = 0.01, Figure 2d). However, this effect was not observed in aortic rings from rats supplemented with dandelion leaf and petal fractions (Figure 2e,f).

**Figure 3:**
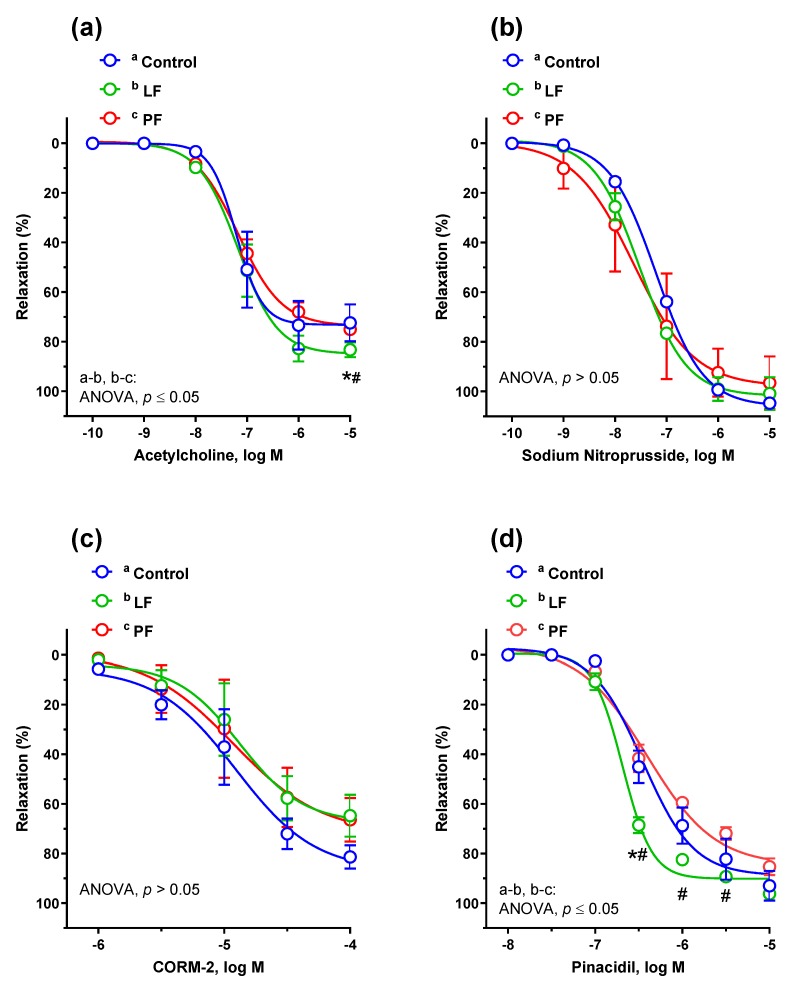
The cumulative concentration-response curves to (**a**) acetylcholine, (**b**) NO donor sodium nitroprusside, (**c**) CO releasing molecule CORM-2 and (**d**) K_ATP_ channel opener pinacidil. From the 8th week of age, rats were supplemented for 4 weeks with either dandelion leaf fraction (LF) or petal fraction (PF). The control group was not supplemented with dandelion. Results (mean ± SEM, *n* = 6) are expressed as a percentage of inhibition of the contraction induced by NA (0.1 μM), *p* ≤ 0.05 (two-way ANOVA/Tukey’s).

**Figure 4:**
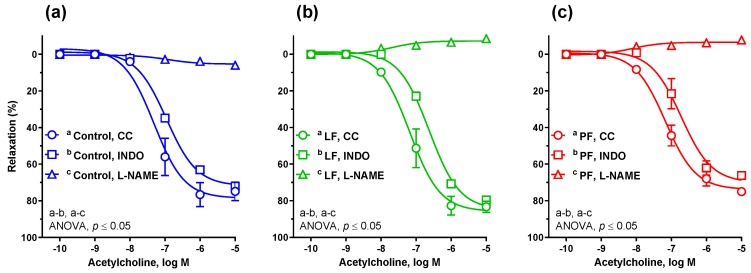
The cumulative concentration-response curves to acetylcholine (**a**) in control rats, (**b**) in rats supplemented with dandelion leaf fraction (LF) and (**c**) in dandelion petal fraction (PF)-fed rats. The aortic rings were preincubated either with nitric oxide synthase inhibitor (l-NAME, 100 μM, 30 min) or the nonselective cyclooxygenase inhibitor (indomethacin, 10 μM, 30 min). Results (mean ± SEM, *n* = 6) are expressed as a percentage of inhibition of the contraction induced by NA (0.1 μM), *p* ≤ 0.05 (two-way ANOVA/Tukey’s). Abbreviations: CC, control conditions; Indo, Indomethacin and l-NAME, N(ω)-nitro-l-arginine methyl ester.

The dandelion leaf fraction potentiated the vasodilator response to the endogenous NO donor ACh, as compared to the control group and to petal fraction (Figure 3a). However, the vascular response to exogenous NO donor SNP and CO donor CORM-2 was similar in all studied groups (Figure 3b,c). The K_ATP_ channel opener pinacidil potentiated the vasodilator response in aortic rings from rats supplemented with leaf fraction, compared to the other two groups (Figure 3d). Preincubation with either l-NAME or indomethacin diminished the vasodilator response to ACh in aortic rings from all studied groups, with no significant difference between the groups (Figure 4a–c).

The E_max_ and pD_2_ parameters are presented in Table 1.

## 4. Discussion

Dandelion fractions have been recognized as a rich source of HCAs, especially l-chicoric acid, with different concentrations in relation to specific plant organs (HCAs: 42% leaf fraction and 21% petal fraction). Previous in vitro and in vivo studies have demonstrated a wide spectrum of actions of dandelion fractions and their components, including antioxidant, anti-inflammatory, anticancer and antimicrobial activities (see Introduction). The beneficial impacts on various cardiovascular parameters in animals fed with a diet enriched with dandelion have also been observed. Firstly, neither a toxic effect nor increased oxidative stress have been revealed by supplementation of the diet of adult female rats with dandelion doses up to 2% [28]. Dandelion alcohol leaf fractions administered to male rats (200–400 mg/kg BW) have been found to possess protective actions against CCl_4_-induced liver tissue toxicity [29]. Moreover, dietary supplementation of rats and rabbits with leaves and roots of dandelions has improved their lipid profiles [22,30]. In another experiment, dandelion water and ethanol fractions (doses up to 3% administered p.o.) decreased the plasma triglyceride, total cholesterol and LDL-C levels in mice fed an atherogenic diet for six weeks [31]. Moreover, dandelion water extract lowered the total cholesterol and triglyceride concentrations, while it increased the serum HDL-C in diabetic rats [32].

In our study, neither blood pressure nor body weight gain were influenced by supplementation with dandelion leaf and petal fractions. The organ weight-to-body weight ratio also did not differ in a significant way. These results confirm previous findings on the lack of dandelion negative influences in the applied concentrations. It is important to note that the dosage of dandelion leaf and petal fractions (11.9 ± 0.6 mg daily) in our experiment delivered daily were 4.92 ± 0.06 mg and 2.57 ± 0.12 mg HCAs, respectively, which are comparable to those usually employed in similar studies [33,34]. Moreover, like the previous researchers [22,30], we have also observed changes in the blood plasma lipid profile of the supplemented rats. Decreased levels in triglycerides, total cholesterol, the lipoprotein combine index and the atherogenic index of plasma induced by leaf fraction were observed.

The two tested dandelion phenolic fractions were shown to exert protective effects in the blood plasma, as measured by biomarkers of oxidative stress: decreased protein carbonylation level (leaf fraction) and an increase in protein thiol groups (petal fraction). These results strengthen the notion of a beneficial role of dandelions as a protective agent against oxidative damage to proteins. On the other hand, neither a strong protective effect against plasma lipid oxidation (TBARS), nor significantly higher ORAC values, were induced by dandelion fractions in our study. These results confirm the findings of our previous in vitro results [3,21]. However, analyzed fractions did exert a protective effect on lipids, measured by the level of decreased TBARS in the spleen (leaf and petal fractions), brain (leaf fraction) and thoracic arteries (petal fraction).

In another set of experiments, we observed that after four weeks of supplementation with dandelion fractions, the contractile response to NA was decreased, which is beneficial on a vasculature. Preincubation with the nonselective COX inhibitor, indomethacin, did not modify the vascular response to NA in aortic rings from rats supplemented with both dandelion fractions, in contrary to the control group. This points to the decreased participation of vasoconstrictor prostanoids in vascular contractions. In a previous study by Hu and Kitts [16], the authors observed the beneficial inhibition of the inflammatory pathway(s) by polyphenol-rich dandelion flower extracts in the mouse arterial wall in vitro, which stays in agreement with decreased TBARS in thoracic arteries of rats supplemented with petal fractions. Supplementation with dandelion leaf fractions increased the aortic response to the K_ATP_ channel opener pinacidil and potentiated vasodilation to acetylcholine (an endogenous NO donor), without modifying the SNP-induced response. These findings point to the upregulation of K_ATP_ channels and increased NO bioavailability in the arteries. In a previous in vivo study, Hu et al. [12] have reported that the vascular endothelium may constitute a possible anti-inflammatory target for dandelion constituents, with an increase in endothelial NO release and/or bioavailability and the enhancement of endothelial NOS expression [35]. This has been confirmed in our study.

Because of the scarcity of in vivo studies on dandelion fractions, further investigations are needed to confirm the benefits and efficacy of dandelion fractions.

## 5. Conclusions

As a result of our in vivo study, we reported changes in the blood plasma lipid profile (leaf fraction), as well as the two selected biomarkers of oxidative stress, expressed by the number of protein thiol groups (petal fraction) and the protein carbonylation level (leaf fraction) in supplemented Wistar rats. Moreover, we observed positive effects on TBARS in the spleen (leaf and petal fractions), brain (leaf fraction) and thoracic arteries (petal fraction), as well as on the carbonyl groups in the liver (leaf vs. petal fraction). The vascular contraction (leaf and petal fractions) and vasodilation (leaf fraction) of the isolated thoracic arteries were also improved.

These results strengthen the concept that there is a wide positive spectrum of activities demonstrated by *T. officinale* fractions and their components, especially hydroxycinnamic acids, and confirm the potential of the *T. officinale* herb as a component of functional foods that could potentially bring a range of benefits for human health.

## Figures and Tables

**Table 1:** Vasodilatory effects of acetylcholine, sodium nitroprusside, carbon monoxide-releasing molecule and pinacidil in the thoracic arteries of experimental rats supplemented with dandelion leaf and petal fractions.

Drug	Control		Leaf Fraction		Petal Fraction	
	E_max_ (%)	pEC_50_	E_max _(%)	pEC_50_	E_max _(%)	pEC_50_
**ACh**	73 ± 5	7.21 ± 0.19	85 ± 4 ^a,b^	7.18 ± 0.10	74 ± 3	7.17 ± 0.08
**+Indomethacin**	71 ± 3	6.97 ± 0.07	80 ± 6	6.70 ± 0.04 *	70 ± 8	6.83 ± 0.17
**+l-NAME**	-	-	-	-	-	-
**SNP**	98 ± 6	7.45 ± 0.18	101 ± 2	7.52 ± 0.27	94 ± 8	7.63 ± 0.27
**CORM-2**	87 ± 15	4.91 ± 0.18	67 ± 13	4.88 ± 0.19	72 ± 25	4.93 ± 0.33
**Pinacidil**	90 ± 6	6.27 ± 0.30	90 ± 2	6.72 ± 0.24 ^a,b^	88 ± 6	6.26 ± 0.41

Acetylcholine-induced vasodilation was analyzed in the absence and presence of the NO synthase inhibitor l-NAME (100 μmol/L, 30 min) and the nonspecific COX inhibitor indomethacin (10 μmol/L, 30 min). Results are expressed as mean ± SEM, *n* = 6. *p* ≤ 0.05 (two-way ANOVA with Tukey’s multiple comparisons test). **^a^** vs. control rats not supplemented with dandelion. **^b^** vs. rats supplemented with petal fraction. * vs. control conditions (comparison with acetylcholine). Abbreviations: ACh, acetylcholine chloride; CORM-2, CO-releasing molecule; COX, cyclooxygenase; NO, nitric oxide and SNP, sodium nitroprusside.

## Supplementary Materials

The following are available online at https://www.mdpi.com/2076-3921/9/2/131/s1, Table S1: The body weight, food intake, systolic blood pressure, organ-to-body weight ratios and pH of caecum in experimental Wistar rats. Table S2: Blood plasma glucose and lipid profile of experimental Wistar rats. Table S3: The antioxidant capacity of experimental Wistar rats.
