# Supplementary material for: Phenolic Fractions from Dandelion Leaves and Petals as Modulators of the Antioxidant Status and Lipid Profile in an In Vivo Study
Source: Antioxidants (Basel). 2020 Feb 3;9(2):131. doi: 10.3390/antiox9020131 (PMC7071135; doi:10.3390/antiox9020131)
Supplement: Supplementary file 1 [file antioxidants-09-00131-s001.pdf]

antioxidants

MDPI

Table S1. The body weight, food intake, systolic blood pressure, organ-to-body weight ratios and pH of caecum in experimental Wistar rats.

	Body weight – begin of feeding (g)	Body weight – end of feeding (g)	Body weight gain[1] (g)	Daily food intake[2] (g)	Daily dandelion intake[3] (mg)	Heart rate (bpm)	Systolic BP (mmHg)	Diastolic BP (mmHg)
**Control (C)**	190 ± 2	324 ± 10	134 ± 8	18.0 ± 0.3	-	414 ± 10	172 ± 32	87 ± 3
	191 (186–193)	326 (306–340)	135 (120–146)	18.1 (17.4– 18.6)	-	414 (403–423)	186 (121–229)	90 (81–91)
**Leaf fraction (LF)**	190 ± 3	308 ± 6	118 ± 3	16.9 ± 0.2	11.7 ± 0.1	383 ± 16	142 ± 29	97 ± 7
	189 (185–197)	310 (298–317)	120 (113–121)	16.9 (16.6–17.2)	11.7 (11.5–12.1)	387 (351–410)	119 (104–201)	91 (90–111)
**Petal fraction (PF)**	190 ± 2	315 ± 14	124 ± 12	17.3 ± 0.8	12.0 ± 0.6	409 ± 26	156 ± 36	112 ± 15
	192 (187–193)	322 (287–335)	130 (100–143)	17.8 (15.7– 18.4)	12.4 (10.9–12.8)	407 (356–465)	120 (118–228)	104 (91–142)
				p*–Value				
**C *vs.* LF**	0.9695	0.2409	0.2516	0.3722	-	0.9916	0.1634	0.9519
**C *vs.* PF**	0.9456	0.6199	0.5364	0.6476	-	0.9916	0.2555	0.9418
**LF *vs.* PF**	0.9936	0.7073	0.7495	0.8343	0.8113	0.9916	0.9963	0.9519

	Organ to body weight ratio (in %)[4]				Intestinal digesta (g)			pH
	**Heart**	**Liver**	**Spleen**	**Brain**	**Filled cecum**	**Empty cecum**	**Part of the small intestine**	**Cecum**
**Control (C)**	0.233 ± 0.007	3.93 ± 0.19	0.21 ± 0.03	0.56 ± 0.02	2.72 ± 0.10	0.71 ± 0.11	4.5 ± 1.5	7.23 ± 0.09
	0.239 (0.219–0.241)	3.77 (3.70–4.31)	0.27 (0.21–0.33)	0.57 (0.53–0.59)	2.68 (2.57–2.90)	0.62 (0.57–0.93)	5.58 (1.63–6.34)	7.20 (7.08–7.40)
**Leaf fraction (LF)**	0.246 ± 0.009	3.60 ± 0.04	0.28 ± 0.04	0.59 ± 0.01	2.49 ± 0.14	0.64 ± 0.07	4.5 ± 1.4	7.07 ± 0.05
	0.249 (0.229–0.260)	3.58 (3.56–3.68)	0.29 (0.21–0.34)	0.58 (0.58–0.62)	2.61 (2.21–2.64)	0.61 (0.54–0.77)	5.88 (1.60–5.97)	7.04 (7.01–7.17)
**Petal fraction (PF)**	0.233 ± 0.003	3.94 ± 0.43	0.29 ± 0.02	0.59 ± 0.03	2.78 ± 0.08	0.55 ± 0.04	4.2 ± 1.2	7.23 ± 0.18
	0.233 (0.227–0.238)	3.56 (3.46–4.79)	0.29 (0.26–0.32)	0.56 (0.55–0.66)	2.74 (2.67–2.94)	0.57 (0.48–0.60)	5.10 (1.89–5.68)	7.29 (6.91–7.51)
				p*–Value				
**C *vs.* LF**	0.3198	0.1760	0.8164	0.2502	0.2486	0.6388	0.9876	0.2260
**C *vs.* PF**	0.9709	0.9802	0.5722	0.5236	0.6436	0.2582	0.8834	0.9712
**LF *vs.* PF**	0.2404	0.4783	0.7968	0.9860	0.1442	0.3194	0.8961	0.4288

Values are expressed as the mean ± SEM and median (with Q1 and Q3) from $n = 6$ rats per each group, $^*p \leq 0.05$ (two-way ANOVA with *Tukey's* multiple comparisons test). N varies among bioassays due to limitations on samples volume collected from animal subjects and/or data outlier detection by the Grubbs' test. Q1 and Q3 are 25th and 75th percentiles. [1] Calculated as: final body weight minus initial body weight. [2] Daily food intake per animal during 28 days of dietary supplementation. [3] Daily dandelion intake (DDI, mg) was calculated as: daily food intake (g) * 694 mg/1000 g. daily HCAs intake (mg) was calculated as: DDI * 420 mg/1000 mg for leaves and DDI * 214 mg/1000 mg for petals; daily L-chicoric acid intake (mg) was calculated as DDI * 350 mg/1000 mg for leaves and DDI * 117 mg/1000 mg for petals. [4] The weight of the internal organs is calculated as: organ weight/final body weight (%). Bold values indicate statistically significant differences. *Abbreviations:* BP, blood pressure; LF, leaf fraction; PF, petal fraction.

Table S2. Blood plasma glucose and lipid profile of experimental Wistar rats.

	Glucose (mmol/L)	Traditional lipid profile (mmol/L)			
		TG	TC	HDL–C	LDL–C
**Control (C)**	17.6 ± 0.9	3.6 ± 0.5	3.2 ± 0.2	0.48 ± 0.03	0.44 ± 0.02
	17.1 (16.1–19.4)	3.1 (3.0–4.6)	3.4 (2.9–3.5)	0.50 (0.42–0.51)	0.42 (0.42–0.47)
**Leaf fraction (LF)**	18.4 ± 0.2	1.6 ± 0.2	2.4 ± 0.4	0.44 ± 0.01	0.39 ± 0.03
	18.5 (18.1–18.7)	1.5 (1.2–2.0)	2.1 (1.9–3.1)	0.45 (0.41–0.46)	0.36 (0.35–0.46)
**Petal fraction (PF)**	18.5 ± 1.3	2.7 ± 0.4	2.9 ± 0.3	0.45 ± 0.05	0.49 ± 0.03
	19.1 (16.0–20.5)	2.7 (2.1–3.4)	2.7 (2.6–3.5)	0.42 (0.38–0.56)	0.48 (0.45–0.54)
		p*–Value			
**C *vs.* LF**	0.8665	**0.0262***	**0.05***	0.7240	0.5270
**C *vs.* PF**	0.8262	0.2634	0.4481	0.8717	0.4487
**LF *vs.* PF**	0.9961	**0.05***	0.2910	0.9552	0.1345

	Nontraditional lipid profile [1]					
	Non–HDL (mmol/L)	TC/HDL–C	LDL–C/HDL–C	AI	LCI	AIP
**Control (C)**	2.8 ± 0.2	6.9 ± 0.7	0.93 ± 0.09	5.9 ± 0.7	11 ± 3.1	0.87 ± 0.09
	2.9 (2.4–3.0)	6.9 (5.7–8.0)	0.84 (0.82–1.12)	5.9 (4.7–7.0)	9.0 (7.1–17.4)	0.79 (0.77–1.04)
**Leaf fraction (LF)**	1.9 ± 0.4	5.4 ± 0.8	0.89 ± 0.07	4.4 ± 0.8	4 ± 1.4	0.54 ± 0.07
	1.7 (1.4–2.7)	5.0 (4.1–7.0)	0.85 (0.78–1.02)	4.0 (3.1–6.0)	2.7 (1.7–6.4)	0.57 (0.40–0.65)
**Petal fraction (PF)**	2.5 ± 0.3	6.7 ± 1.1	1.11 ± 0.13	5.7 ± 1.1	10 ± 3.1	0.78 ± 0.10
	2.3 (2.1–3.1)	7.0 (4.8–8.4)	1.18 (0.86–1.29)	6.0 (3.8–7.4)	8.6 (4.7–15.3)	0.86 (0.57–0.90)
			p*–Value			
**C *vs.* LF**	0.2316	0.5279	0.9574	0.5279	**0.05***	**0.0449***
**C *vs.* PF**	0.8194	0.9931	0.4942	0.9931	0.8840	0.7597
**LF *vs.* PF**	0.4371	0.5846	0.3735	0.5846	0.2863	0.2446

Values are expressed as the mean ± SEM and median (with Q1 and Q3) from $n = 6$ rats per each group, *$p \leq 0.05$ (two-way ANOVA with *Tukey's* multiple comparisons test). N varies among bioassays due to limitations on samples volume collected from animal subjects and/or data outlier detection by the Grubbs' test. Q1 and Q3 are 25th and 75th percentiles. [1] A nontraditional lipid profile was calculated as non-HDL-C: TC minus HDL-C; LDL-C/HDL-C; AI, non-HDL-C/HDL-C; LCI, TC∗TG∗LDL-C/HDL-C; AIP, log10(TG/HDL-C). Bold values indicate statistically significant differences. *Abbreviations:* AI, atherogenic index; AIP, atherogenic index of plasma; HDL-C, high-density lipoprotein cholesterol; LCI, lipoprotein combine index; LDL-C, low-density lipoprotein cholesterol; LF, leaf fraction; PF, petal fraction; TC, total cholesterol; TG, triglyceride.

Table S3. The antioxidant capacity of experimental Wistar rats.

	Thiol groups (nmol/mL/mg of protein)	Carbonyl groups (nmol/mL/mg of protein or nmol/mg homogenate)					F2α–isoprostane
	**Plasma**	**Plasma**	**Heart**	**Liver**	**Spleen**	**Brain**	**Urine**
**Control (C)**	60 ± 6<br>55 (53–73)	29.2 ± 0.2<br>29.1 (29–29.6)	1.5 ± 0.3<br>1.66 (0.99–1.9)	12.3 ± 2.5<br>11.0 (8.8–17.0)	36 ± 3<br>37.8 (29.6–39.4)	18.6 ± 2.4<br>21.0 (13.7–21.2)	20 ± 8<br>14 (9.4–34.9)
**Leaf fraction (LF)**	81 ± 12<br>79 (62–103)	17.2 ± 1.8<br>15.73 (15.1–20.9)	1.9 ± 0.3<br>1.85 (1.28–2.46)	19.2 ± 1.9<br>18.6 (17.9–24.0)	49 ± 13<br>54.3 (24.6–68.7)	23.1 ± 3.3<br>20.9 (18.9–29.5)	18 ± 9<br>11 (7.8–36.1)
**Petal fraction (PF)**	96 ± 3<br>97 (90–100)	29.0 ± 5.3<br>29.3 (19.6–38.2)	1.5 ± 0.1<br>1.53 (1.31–1.76)	10.9 ± 3.6<br>9.6 (4.9–15.1)	31± 7<br>26.0 (21.3–45.3)	25.2 ± 3.6<br>23.3 (20.1–32.1)	14 ± 3<br>12 (10.4–19.0)
	p*–Value						
**C *vs*. LF**	0.2033	**0.0214***	0.3636	0.0646	0.4055	0.3867	0.9074
**C *vs*. PF**	**0.0076***	0.9902	0.9986	0.4772	0.8761	0.1907	0.5025
**LF *vs*. PF**	0.3093	**0.0486***	0.3825	**0.0486***	0.2424	0.7860	0.6509
	**ORAC (μmol TE/L)**	**TBARS (nmol/mL or nmol/mg homogenate)**					
	**Plasma**	**Plasma**	**Thoracic artery**	**Heart**	**Liver**	**Spleen**	**Brain**
**Control (C)**	578 ± 2<br>579 (574–581)	1.32 ± 0.02<br>1.33 (1.28–1.36)	0.18 ± 0.04<br>0.18 (0.11–0.25)	0.23 ± 0.06<br>0.20 (0.14–0.34)	0.120 ± 0.018<br>0.122 (0.088–0.150)	0.408 ± 0.023<br>0.412 (0.365–0.446)	0.058 ± 0.006<br>0.064 (0.045–0.066)
**Leaf fraction (LF)**	573 ± 13<br>571 (551–596)	1.70 ± 0.23<br>1.76 (1.29–2.06)	0.13 ± 0.03<br>0.13 (0.07–0.18)	0.14 ± 0.05<br>0.10 (0.09–0.24)	0.127 ± 0.007<br>0.130 (0.113–0.137)	0.317 ± 0.006<br>0.314 (0.308–0.330)	0.031 ± 0.003<br>0.028 (0.028–0.038)
**Petal fraction (PF)**	627 ± 24<br>649 (578–653)	1.36 ± 0.03<br>1.35 (1.33–1.42)	0.10 ± 0.04<br>0.08 (0.05–0.18)	0.15 ± 0.02<br>0.15 (0.11–0.19)	0.121 ± 0.012<br>0.111 (0.107–0.145)	0.327 ± 0.012<br>0.327 (0.306–0.348)	0.036 ± 0.012<br>0.043 (0.013–0.051)
	p*–Value						
**C *vs*. LF**	0.9612	0.1706	**0.05***	0.5827	0.7719	**0.0208***	**0.0225***
**C *vs*. PF**	0.1183	0.9651	**0.05***	0.6161	0.9938	**0.0379***	0.1651
**LF *vs*. PF**	0.0910	0.2222	0.7057	0.9978	0.8266	0.5219	0.7370

Values are expressed as the mean ± SEM and median (with Q1 and Q3) from $n = 6$ rats per each group, $*p \leq 0.05$ (two-way ANOVA with *Tukey's* multiple comparisons test). N varies among bioassays due to limitations on samples volume collected from animal subjects and/or data outlier detection by the Grubbs' test. Q1 and Q3 are 25th and 75th percentiles. Bold values indicate statistically significant differences. *Abbreviations:* LF, leaf fraction; PF, petal fraction.
